# Lurasidone hydro­chloride

**DOI:** 10.1107/S1600536812012883

**Published:** 2012-04-13

**Authors:** Hua Zhang, Hubo Wang, Xueyan Zhu, Zhedong Yuan, Huijuan Jiang

**Affiliations:** a Shanghai Institute of Pharmaceutical Industry, No. 1111, North ZhongShan No.1 Road, HongKou District, Shanghai 200437, People’s Republic of China

## Abstract

In the crystal structure of the title compound, C_28_H_37_N_4_O_2_S^+^·Cl^−^ [systematic name: 4-(1,2-benzothia­zol-3-yl)-1-({2-[(3,5-dioxo-4-aza­tricyclo­[5.2.1.0^2,6^]decan-4-yl)meth­yl]cyclo­hex­yl}meth­yl)piperazin-1-ium chloride], the anions and cations are linked by N—H⋯Cl hydrogen bonds. The crystal structure is further stabilized by C—H⋯π and C—H⋯O inter­actions.

## Related literature

For the background to the biological activity of the title compound, an anti­psychotic drug, see: Ishibashi *et al.* (2002 ▶); Ishiyama *et al.* (2003 ▶); Ohno *et al.* (1997 ▶).
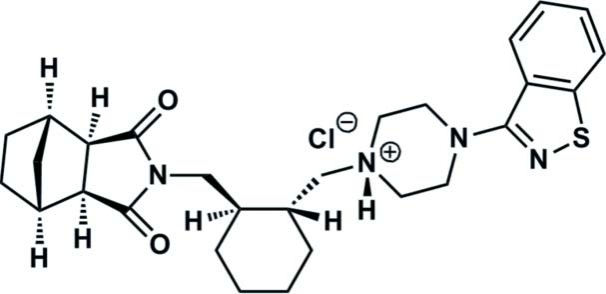


## Experimental

### 

#### Crystal data

C_28_H_37_N_4_O_2_S^+^·Cl^−^
*M* *_r_* = 529.13
Orthorhombic, 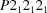

*a* = 11.2039 (10) Å
*b* = 12.2665 (11) Å
*c* = 19.9774 (18) Å
*V* = 2745.5 (4) Å^3^
*Z* = 4
Mo *K*α radiation
μ = 0.25 mm^−1^
*T* = 293 K
0.22 × 0.12 × 0.10 mm

#### Data collection

Bruker SMART CCD area-detector diffractometer
Absorption correction: multi-scan (*SADABS*; Bruker, 2000 ▶) *T*_min_ = 0.452, *T*_max_ = 1.000
15020 measured reflections
5384 independent reflections
4649 reflections with *I* > 2σ(*I*)
*R*_int_ = 0.028

#### Refinement

*R*[*F* ^2^ > 2σ(*F* ^2^)] = 0.047
*wR*(*F* ^2^) = 0.108
*S* = 1.10
5384 reflections
329 parameters
1 restraint
H atoms treated by a mixture of independent and constrained refinement
Δρ_max_ = 0.24 e Å^−3^
Δρ_min_ = −0.14 e Å^−3^
Absolute structure: Flack (1983 ▶), 2338 Friedel pairs
Flack parameter: 0.03 (6)

### 

Data collection: *SMART* (Bruker, 2000 ▶); cell refinement: *SAINT* (Bruker, 2000 ▶); data reduction: *SAINT*; program(s) used to solve structure: *SHELXS97* (Sheldrick, 2008 ▶); program(s) used to refine structure: *SHELXL97* (Sheldrick, 2008 ▶); molecular graphics: *SHELXTL* (Sheldrick, 2008 ▶); software used to prepare material for publication: *SHELXTL*.

## Supplementary Material

Crystal structure: contains datablock(s) I, global. DOI: 10.1107/S1600536812012883/bt5835sup1.cif

Supplementary material file. DOI: 10.1107/S1600536812012883/bt5835Isup2.cml

Structure factors: contains datablock(s) I. DOI: 10.1107/S1600536812012883/bt5835Isup3.hkl

Additional supplementary materials:  crystallographic information; 3D view; checkCIF report

## Figures and Tables

**Table 1:** Hydrogen-bond geometry (Å, °) *Cg* is the centroid of the S1/ N4/C22/C23/C28 ring.

*D*—H⋯*A*	*D*—H	H⋯*A*	*D*⋯*A*	*D*—H⋯*A*
N2—H2*A*⋯Cl1	0.80 (2)	2.15 (2)	2.9426 (19)	168 (2)
C21—H21*A*⋯O1^i^	0.97	2.38	3.289 (3)	156
C5—H5*A*⋯*Cg*^ii^	0.97	2.89	3.802 (4)	157

Symmetry codes: (i) 
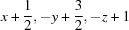
; (ii) 
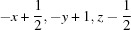
.

**Fig. 1.**
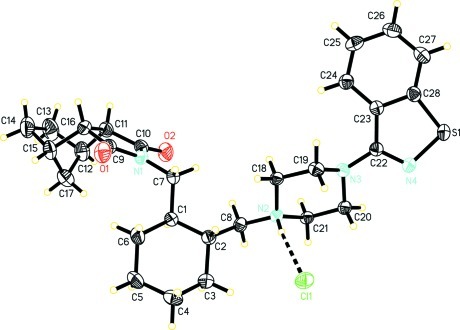
The molecular structure of lurasidone hydrochloride, showing the atom-numbering scheme. Displacement ellipsoids are drawn at the 30% probability level.

**Fig. 2.**
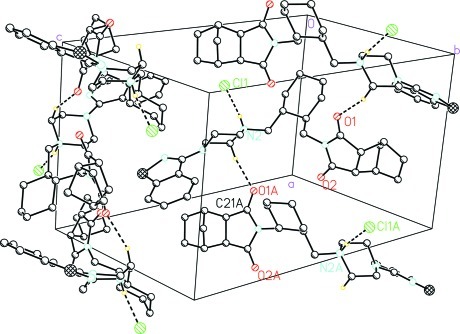
Part of the crystal packing of the title compound, showing the N—H···Cl and C—H···O hydrogen bonds. Hydrogen bonds are shown as dashed lines and H atoms not involved in hydrogen bonding have been omitted for clarity. Only atoms involved in hydrogen bonding have been labelled.

## supplementary crystallographic information

### Comment

Lurasidone hydrochloride is a benzisothiazole derivative and an atypical antipsychotic drug. This drug has high affinities for dopamine D_2_ (K_i_ =1.68nM), serotonin 5-HT_2 A_ (K_i_ =2.03nM), 5-HT_1 A_ (K_i_ =6.75nM), 5-HT_7_ receptors (K_i_ =0.495nM), and α_2c_ adrenoceptor (K_i_ =10.8nM), but only weak or negligible interactions with serotonin 5-HT_2c_, histamine H_1_, acetylcholine *M*_1_ receptors, and α_1_ adrenoceptor (Ishibashi *et al.*, 2002; Ishiyama *et al.*, 2003). Interestingly, despite its potent D_2_-blocking actions *in vivo*, Lurasidone has little propensity to induce extrapyramidal symptoms (Ohno *et al.*, 1997).

The United States Food and Drug Administration (US FDA) approved Lurasidone hydrochloride (brand name: Latuda) in 2010 as an immediate release oral tablet for the treatment of schizophrenia. Lurasidone hydrochloride was developed by Dainippon Sumitomo Pharma in collaboration with Merck Research Laboratories during the initial IND stages. No data about crystal structure of Lurasidone hydrochloride has been reported yet.

Lurasidone hydrochloride consists of six chiral centres, e. g. C1, C2, C11, C12, C15 and C16. Currently, the clinically used form is a single isomer. The crystal structure of the title compound is built up of discrete lurasidium anions and chloride cations (Fig. 1).

There are two systems of hydrogen-bond interactions, *viz.* N2—H2A···Cl1 and C21—H21A···O1A^i^ [symmetry code: (i) *x* + 1/2, -*y* + 3/2, -*z* + 1] (Table 1 and Fig. 2).

The crystal structure is further stabilized by weak C—H···π interactions [symmetry code: 1/2 - *x*, 1 - *y*, -1/2 + *z*] between the cyclohexyl (C5—H5A) and the isothiazole ring.

### Experimental

Lurasidone hydrochloride (10 g, 18.9 mmole) was dissolved in a hot solution in a 3:1 mixture of acetone and water. After cooling to ambient temperature, the solvent was allowed to evaporate slowly. Colourless crystals of (1) appeared after 5 days.

### Refinement

The coordinates of the N-bonded H-atom were refined with the N-H distance restrained to = 0.80 (2) Å. *U*_iso_(H) was set to 1.2*U*_eq_(N). H atoms attached to C atoms were positioned geometrically and treated as riding on their parent C atoms, with C—H = 0.97 Å and, *U*_iso_(H) = 1.2*U*_eq_(C) for methylene, C—H = 0.98 Å and *U*_iso_ = 1.2*U*_eq_(C) for *tert*-methyl, and C—H = 0.93 Å and *U*_iso_ = 1.2*U*_eq_(C) for aromatic H atoms.

### Crystal data

**Table:**

C_28_H_37_N_4_O_2_S^+^·Cl^−^	*F*(000) = 1128
*M**_r_* = 529.13	*D*_x_ = 1.280 Mg m^−^^3^
Orthorhombic, *P*2_1_2_1_2_1_	Mo *K*α radiation, λ = 0.71073 Å
Hall symbol: P 2ac 2ab	Cell parameters from 3969 reflections
*a* = 11.2039 (10) Å	θ = 4.9–45.2°
*b* = 12.2665 (11) Å	µ = 0.25 mm^−^^1^
*c* = 19.9774 (18) Å	*T* = 293 K
*V* = 2745.5 (4) Å^3^	Prismatic, colourless
*Z* = 4	0.22 × 0.12 × 0.10 mm

### Data collection

**Table:**

Bruker SMART CCD area-detector diffractometer	5384 independent reflections
Radiation source: fine-focus sealed tube	4649 reflections with *I* > 2σ(*I*)
Graphite monochromator	*R*_int_ = 0.028
phi and ω scans	θ_max_ = 26.0°, θ_min_ = 2.0°
Absorption correction: multi-scan (*SADABS*; Bruker, 2000)	*h* = −13→13
*T*_min_ = 0.452, *T*_max_ = 1.000	*k* = −15→15
15020 measured reflections	*l* = −24→13

### Refinement

**Table:**

Refinement on *F*^2^	Secondary atom site location: difference Fourier map
Least-squares matrix: full	Hydrogen site location: inferred from neighbouring sites
*R*[*F*^2^ > 2σ(*F*^2^)] = 0.047	H atoms treated by a mixture of independent and constrained refinement
*wR*(*F*^2^) = 0.108	*w* = 1/[σ^2^(*F*_o_^2^) + (0.0569*P*)^2^ + 0.004*P*] where *P* = (*F*_o_^2^ + 2*F*_c_^2^)/3
*S* = 1.10	(Δ/σ)_max_ = 0.001
5384 reflections	Δρ_max_ = 0.24 e Å^−^^3^
329 parameters	Δρ_min_ = −0.14 e Å^−^^3^
1 restraint	Absolute structure: Flack (1983), 2338 Friedel pairs
Primary atom site location: structure-invariant direct methods	Flack parameter: 0.03 (6)

### Special details

**Table:**

Geometry. All e.s.d.'s (except the e.s.d. in the dihedral angle between two l.s. planes) are estimated using the full covariance matrix. The cell e.s.d.'s are taken into account individually in the estimation of e.s.d.'s in distances, angles and torsion angles; correlations between e.s.d.'s in cell parameters are only used when they are defined by crystal symmetry. An approximate (isotropic) treatment of cell e.s.d.'s is used for estimating e.s.d.'s involving l.s. planes.
Refinement. Refinement of *F*^2^ against ALL reflections. The weighted *R*-factor *wR* and goodness of fit *S* are based on *F*^2^, conventional *R*-factors *R* are based on *F*, with *F* set to zero for negative *F*^2^. The threshold expression of *F*^2^ > σ(*F*^2^) is used only for calculating *R*-factors(gt) *etc*. and is not relevant to the choice of reflections for refinement. *R*-factors based on *F*^2^ are statistically about twice as large as those based on *F*, and *R*- factors based on ALL data will be even larger.

### Fractional atomic coordinates and isotropic or equivalent isotropic displacement parameters (Å^2^)

**Table:**

	*x*	*y*	*z*	*U*_iso_*/*U*_eq_	
S1	0.68064 (7)	0.23143 (5)	0.79887 (4)	0.0593 (2)	
Cl1	0.24826 (6)	0.28761 (6)	0.52740 (4)	0.0704 (2)	
N1	0.43096 (19)	0.85877 (16)	0.44646 (11)	0.0484 (5)	
N2	0.44531 (15)	0.44620 (14)	0.52716 (10)	0.0353 (4)	
N3	0.54606 (16)	0.38440 (15)	0.65254 (10)	0.0406 (5)	
N4	0.60954 (19)	0.24766 (16)	0.72566 (11)	0.0499 (5)	
O1	0.25234 (18)	0.94287 (16)	0.46631 (10)	0.0662 (5)	
O2	0.62622 (17)	0.81973 (17)	0.42244 (12)	0.0748 (6)	
C1	0.3506 (2)	0.67528 (19)	0.41447 (13)	0.0455 (6)	
H1	0.4094	0.6737	0.3782	0.055*	
C2	0.3346 (2)	0.55930 (19)	0.44089 (12)	0.0438 (6)	
H2	0.2824	0.5628	0.4802	0.053*	
C3	0.2738 (3)	0.4859 (2)	0.38862 (17)	0.0687 (9)	
H3A	0.3267	0.4770	0.3506	0.082*	
H3B	0.2603	0.4144	0.4080	0.082*	
C4	0.1544 (3)	0.5325 (3)	0.36440 (19)	0.0818 (10)	
H4A	0.1199	0.4846	0.3310	0.098*	
H4B	0.0992	0.5376	0.4017	0.098*	
C5	0.1745 (3)	0.6445 (3)	0.33458 (17)	0.0754 (9)	
H5A	0.0987	0.6750	0.3204	0.090*	
H5B	0.2256	0.6385	0.2956	0.090*	
C6	0.2325 (2)	0.7196 (2)	0.38630 (15)	0.0636 (8)	
H6A	0.1771	0.7307	0.4230	0.076*	
H6B	0.2473	0.7900	0.3658	0.076*	
C7	0.3973 (2)	0.75009 (19)	0.46978 (14)	0.0516 (6)	
H7A	0.3362	0.7574	0.5039	0.062*	
H7B	0.4663	0.7161	0.4904	0.062*	
C8	0.4524 (2)	0.50733 (18)	0.46260 (13)	0.0429 (6)	
H8A	0.4788	0.4578	0.4278	0.052*	
H8B	0.5121	0.5641	0.4669	0.052*	
C9	0.3553 (2)	0.9473 (2)	0.44790 (14)	0.0510 (6)	
C10	0.5450 (2)	0.8844 (2)	0.42461 (14)	0.0543 (7)	
C11	0.5473 (2)	1.0026 (2)	0.40424 (16)	0.0586 (7)	
H11	0.6089	1.0429	0.4288	0.070*	
C12	0.5566 (3)	1.0230 (3)	0.32838 (18)	0.0724 (9)	
H12	0.6171	0.9793	0.3053	0.087*	
C13	0.5667 (3)	1.1452 (3)	0.3173 (2)	0.0956 (13)	
H13A	0.5866	1.1616	0.2711	0.115*	
H13B	0.6269	1.1768	0.3463	0.115*	
C14	0.4411 (3)	1.1879 (3)	0.3352 (2)	0.0903 (11)	
H14A	0.4442	1.2381	0.3727	0.108*	
H14B	0.4043	1.2241	0.2972	0.108*	
C15	0.3741 (3)	1.0839 (2)	0.35359 (16)	0.0643 (8)	
H15	0.2870	1.0896	0.3507	0.077*	
C16	0.4211 (2)	1.0450 (2)	0.42160 (14)	0.0555 (7)	
H16	0.4237	1.1043	0.4544	0.067*	
C17	0.4291 (3)	1.0019 (3)	0.30617 (16)	0.0728 (9)	
H17A	0.4158	1.0201	0.2595	0.087*	
H17B	0.4033	0.9279	0.3151	0.087*	
C18	0.4400 (2)	0.51847 (18)	0.58658 (12)	0.0404 (5)	
H18A	0.3685	0.5629	0.5843	0.049*	
H18B	0.5085	0.5668	0.5865	0.049*	
C19	0.4391 (2)	0.4532 (2)	0.65033 (12)	0.0446 (6)	
H19A	0.4380	0.5018	0.6886	0.054*	
H19B	0.3681	0.4079	0.6521	0.054*	
C20	0.5448 (2)	0.30604 (19)	0.59814 (12)	0.0417 (5)	
H20A	0.4737	0.2611	0.6010	0.050*	
H20B	0.6141	0.2589	0.6012	0.050*	
C21	0.54609 (19)	0.36631 (17)	0.53267 (12)	0.0387 (5)	
H21A	0.6213	0.4049	0.5282	0.046*	
H21B	0.5407	0.3142	0.4963	0.046*	
C22	0.5904 (2)	0.35074 (19)	0.71390 (12)	0.0396 (5)	
C23	0.63088 (19)	0.42610 (19)	0.76474 (12)	0.0395 (5)	
C24	0.6322 (2)	0.5409 (2)	0.76601 (13)	0.0473 (6)	
H24	0.6003	0.5809	0.7307	0.057*	
C25	0.6813 (2)	0.5925 (2)	0.82006 (15)	0.0597 (7)	
H25	0.6816	0.6682	0.8215	0.072*	
C26	0.7306 (3)	0.5342 (3)	0.87282 (15)	0.0638 (8)	
H26	0.7618	0.5716	0.9093	0.077*	
C27	0.7342 (3)	0.4226 (3)	0.87204 (14)	0.0601 (7)	
H27	0.7692	0.3837	0.9068	0.072*	
C28	0.6836 (2)	0.3692 (2)	0.81747 (12)	0.0467 (6)	
H2A	0.3847 (16)	0.4111 (16)	0.5276 (12)	0.037 (6)*	

### Atomic displacement parameters (Å^2^)

**Table:**

	*U*^11^	*U*^22^	*U*^33^	*U*^12^	*U*^13^	*U*^23^
S1	0.0766 (5)	0.0453 (3)	0.0561 (4)	−0.0030 (3)	−0.0137 (4)	0.0137 (3)
Cl1	0.0561 (4)	0.0614 (4)	0.0938 (6)	−0.0249 (3)	−0.0178 (4)	0.0066 (4)
N1	0.0479 (11)	0.0405 (11)	0.0569 (13)	0.0061 (9)	−0.0117 (10)	−0.0027 (10)
N2	0.0285 (9)	0.0358 (10)	0.0417 (11)	−0.0027 (8)	0.0012 (8)	−0.0007 (9)
N3	0.0380 (10)	0.0405 (11)	0.0432 (12)	0.0105 (8)	−0.0033 (9)	−0.0032 (8)
N4	0.0570 (12)	0.0400 (11)	0.0526 (13)	−0.0056 (9)	−0.0067 (10)	0.0059 (9)
O1	0.0633 (12)	0.0681 (12)	0.0673 (13)	0.0200 (10)	0.0118 (11)	0.0036 (10)
O2	0.0536 (11)	0.0732 (14)	0.0975 (17)	0.0233 (10)	−0.0105 (11)	0.0050 (12)
C1	0.0471 (13)	0.0446 (13)	0.0449 (14)	0.0025 (11)	−0.0076 (11)	0.0008 (11)
C2	0.0406 (12)	0.0459 (13)	0.0450 (14)	−0.0031 (11)	−0.0008 (11)	0.0026 (11)
C3	0.076 (2)	0.0525 (16)	0.077 (2)	−0.0086 (14)	−0.0293 (17)	0.0018 (14)
C4	0.073 (2)	0.082 (2)	0.091 (2)	−0.0179 (18)	−0.0384 (18)	0.0016 (19)
C5	0.0680 (19)	0.081 (2)	0.077 (2)	−0.0042 (17)	−0.0353 (17)	0.0109 (18)
C6	0.0590 (16)	0.0590 (17)	0.073 (2)	0.0048 (14)	−0.0211 (15)	0.0119 (15)
C7	0.0556 (14)	0.0452 (14)	0.0540 (16)	0.0078 (11)	−0.0112 (13)	0.0038 (12)
C8	0.0435 (13)	0.0404 (13)	0.0449 (15)	−0.0001 (10)	0.0051 (11)	0.0032 (11)
C9	0.0523 (15)	0.0498 (15)	0.0510 (16)	0.0121 (12)	−0.0094 (12)	−0.0058 (12)
C10	0.0453 (14)	0.0578 (17)	0.0598 (18)	0.0073 (13)	−0.0163 (13)	−0.0028 (13)
C11	0.0479 (14)	0.0548 (17)	0.073 (2)	−0.0038 (12)	−0.0193 (14)	0.0060 (14)
C12	0.0573 (17)	0.081 (2)	0.079 (2)	0.0114 (16)	0.0023 (16)	0.0167 (18)
C13	0.075 (2)	0.103 (3)	0.109 (3)	−0.016 (2)	−0.015 (2)	0.045 (2)
C14	0.104 (3)	0.073 (2)	0.094 (3)	0.009 (2)	−0.015 (2)	0.029 (2)
C15	0.0517 (15)	0.0691 (18)	0.072 (2)	0.0122 (15)	−0.0086 (15)	0.0186 (16)
C16	0.0628 (16)	0.0418 (14)	0.0618 (18)	0.0080 (12)	−0.0131 (14)	−0.0058 (13)
C17	0.071 (2)	0.090 (2)	0.058 (2)	−0.0015 (17)	−0.0110 (16)	0.0081 (17)
C18	0.0399 (12)	0.0355 (12)	0.0459 (14)	0.0080 (10)	−0.0045 (11)	−0.0074 (10)
C19	0.0405 (12)	0.0501 (14)	0.0433 (14)	0.0078 (11)	0.0016 (11)	−0.0080 (11)
C20	0.0405 (12)	0.0369 (12)	0.0477 (14)	0.0077 (10)	−0.0036 (11)	−0.0036 (10)
C21	0.0340 (11)	0.0353 (11)	0.0467 (14)	0.0041 (9)	−0.0013 (10)	−0.0067 (11)
C22	0.0360 (11)	0.0403 (12)	0.0425 (14)	−0.0020 (10)	0.0006 (10)	0.0016 (11)
C23	0.0355 (11)	0.0440 (13)	0.0389 (13)	0.0004 (10)	0.0052 (10)	0.0013 (10)
C24	0.0447 (13)	0.0444 (13)	0.0526 (16)	0.0072 (11)	−0.0003 (12)	−0.0017 (12)
C25	0.0617 (16)	0.0456 (14)	0.072 (2)	0.0017 (13)	0.0015 (15)	−0.0155 (13)
C26	0.0681 (19)	0.071 (2)	0.0529 (18)	0.0024 (16)	−0.0095 (15)	−0.0162 (15)
C27	0.0636 (17)	0.0725 (19)	0.0441 (16)	0.0036 (15)	−0.0081 (13)	0.0008 (14)
C28	0.0467 (13)	0.0503 (14)	0.0431 (14)	−0.0043 (11)	0.0011 (12)	0.0052 (11)

### Geometric parameters (Å, º)

**Table:**

S1—N4	1.677 (2)	C11—C12	1.539 (5)
S1—C28	1.731 (3)	C11—C16	1.546 (4)
Cl1—H2A	2.152 (16)	C11—H11	0.9800
N1—C9	1.378 (3)	C12—C17	1.518 (4)
N1—C10	1.386 (3)	C12—C13	1.519 (4)
N1—C7	1.462 (3)	C12—H12	0.9800
N2—C18	1.483 (3)	C13—C14	1.543 (5)
N2—C8	1.494 (3)	C13—H13A	0.9700
N2—C21	1.499 (3)	C13—H13B	0.9700
N2—H2A	0.804 (15)	C14—C15	1.526 (4)
N3—C22	1.386 (3)	C14—H14A	0.9700
N3—C20	1.451 (3)	C14—H14B	0.9700
N3—C19	1.466 (3)	C15—C17	1.512 (4)
N4—C22	1.304 (3)	C15—C16	1.533 (4)
O1—C9	1.212 (3)	C15—H15	0.9800
O2—C10	1.208 (3)	C16—H16	0.9800
C1—C2	1.528 (3)	C17—H17A	0.9700
C1—C7	1.529 (3)	C17—H17B	0.9700
C1—C6	1.537 (3)	C18—C19	1.505 (4)
C1—H1	0.9800	C18—H18A	0.9700
C2—C8	1.528 (3)	C18—H18B	0.9700
C2—C3	1.538 (4)	C19—H19A	0.9700
C2—H2	0.9800	C19—H19B	0.9700
C3—C4	1.533 (4)	C20—C21	1.502 (3)
C3—H3A	0.9700	C20—H20A	0.9700
C3—H3B	0.9700	C20—H20B	0.9700
C4—C5	1.513 (4)	C21—H21A	0.9700
C4—H4A	0.9700	C21—H21B	0.9700
C4—H4B	0.9700	C22—C23	1.446 (3)
C5—C6	1.530 (4)	C23—C28	1.395 (3)
C5—H5A	0.9700	C23—C24	1.409 (3)
C5—H5B	0.9700	C24—C25	1.367 (4)
C6—H6A	0.9700	C24—H24	0.9300
C6—H6B	0.9700	C25—C26	1.389 (4)
C7—H7A	0.9700	C25—H25	0.9300
C7—H7B	0.9700	C26—C27	1.369 (4)
C8—H8A	0.9700	C26—H26	0.9300
C8—H8B	0.9700	C27—C28	1.392 (4)
C9—C16	1.501 (4)	C27—H27	0.9300
C10—C11	1.507 (4)		
			
N4—S1—C28	94.62 (11)	C13—C12—H12	114.9
C9—N1—C10	113.2 (2)	C11—C12—H12	114.9
C9—N1—C7	123.6 (2)	C12—C13—C14	103.5 (3)
C10—N1—C7	123.0 (2)	C12—C13—H13A	111.1
C18—N2—C8	113.15 (17)	C14—C13—H13A	111.1
C18—N2—C21	111.25 (17)	C12—C13—H13B	111.1
C8—N2—C21	110.59 (17)	C14—C13—H13B	111.1
C18—N2—H2A	106.1 (17)	H13A—C13—H13B	109.0
C8—N2—H2A	108.8 (17)	C15—C14—C13	102.7 (3)
C21—N2—H2A	106.6 (16)	C15—C14—H14A	111.2
C22—N3—C20	117.95 (18)	C13—C14—H14A	111.2
C22—N3—C19	119.43 (19)	C15—C14—H14B	111.2
C20—N3—C19	110.51 (18)	C13—C14—H14B	111.2
C22—N4—S1	110.51 (17)	H14A—C14—H14B	109.1
C2—C1—C7	110.4 (2)	C17—C15—C14	101.8 (3)
C2—C1—C6	110.8 (2)	C17—C15—C16	102.0 (2)
C7—C1—C6	110.3 (2)	C14—C15—C16	107.8 (3)
C2—C1—H1	108.4	C17—C15—H15	114.6
C7—C1—H1	108.4	C14—C15—H15	114.6
C6—C1—H1	108.4	C16—C15—H15	114.6
C1—C2—C8	112.67 (19)	C9—C16—C15	113.0 (2)
C1—C2—C3	111.3 (2)	C9—C16—C11	105.0 (2)
C8—C2—C3	109.3 (2)	C15—C16—C11	102.7 (2)
C1—C2—H2	107.8	C9—C16—H16	111.9
C8—C2—H2	107.8	C15—C16—H16	111.9
C3—C2—H2	107.8	C11—C16—H16	111.9
C4—C3—C2	112.5 (3)	C15—C17—C12	95.0 (3)
C4—C3—H3A	109.1	C15—C17—H17A	112.7
C2—C3—H3A	109.1	C12—C17—H17A	112.7
C4—C3—H3B	109.1	C15—C17—H17B	112.7
C2—C3—H3B	109.1	C12—C17—H17B	112.7
H3A—C3—H3B	107.8	H17A—C17—H17B	110.2
C5—C4—C3	109.5 (3)	N2—C18—C19	111.08 (18)
C5—C4—H4A	109.8	N2—C18—H18A	109.4
C3—C4—H4A	109.8	C19—C18—H18A	109.4
C5—C4—H4B	109.8	N2—C18—H18B	109.4
C3—C4—H4B	109.8	C19—C18—H18B	109.4
H4A—C4—H4B	108.2	H18A—C18—H18B	108.0
C4—C5—C6	110.1 (3)	N3—C19—C18	109.03 (19)
C4—C5—H5A	109.6	N3—C19—H19A	109.9
C6—C5—H5A	109.6	C18—C19—H19A	109.9
C4—C5—H5B	109.6	N3—C19—H19B	109.9
C6—C5—H5B	109.6	C18—C19—H19B	109.9
H5A—C5—H5B	108.1	H19A—C19—H19B	108.3
C5—C6—C1	113.6 (2)	N3—C20—C21	109.03 (18)
C5—C6—H6A	108.9	N3—C20—H20A	109.9
C1—C6—H6A	108.9	C21—C20—H20A	109.9
C5—C6—H6B	108.9	N3—C20—H20B	109.9
C1—C6—H6B	108.9	C21—C20—H20B	109.9
H6A—C6—H6B	107.7	H20A—C20—H20B	108.3
N1—C7—C1	113.9 (2)	N2—C21—C20	112.22 (18)
N1—C7—H7A	108.8	N2—C21—H21A	109.2
C1—C7—H7A	108.8	C20—C21—H21A	109.2
N1—C7—H7B	108.8	N2—C21—H21B	109.2
C1—C7—H7B	108.8	C20—C21—H21B	109.2
H7A—C7—H7B	107.7	H21A—C21—H21B	107.9
N2—C8—C2	114.13 (19)	N4—C22—N3	120.5 (2)
N2—C8—H8A	108.7	N4—C22—C23	116.2 (2)
C2—C8—H8A	108.7	N3—C22—C23	122.9 (2)
N2—C8—H8B	108.7	C28—C23—C24	118.8 (2)
C2—C8—H8B	108.7	C28—C23—C22	110.1 (2)
H8A—C8—H8B	107.6	C24—C23—C22	130.9 (2)
O1—C9—N1	123.8 (3)	C25—C24—C23	118.8 (2)
O1—C9—C16	127.5 (2)	C25—C24—H24	120.6
N1—C9—C16	108.7 (2)	C23—C24—H24	120.6
O2—C10—N1	123.8 (3)	C24—C25—C26	121.4 (2)
O2—C10—C11	127.6 (3)	C24—C25—H25	119.3
N1—C10—C11	108.6 (2)	C26—C25—H25	119.3
C10—C11—C12	115.1 (3)	C27—C26—C25	121.2 (3)
C10—C11—C16	104.3 (2)	C27—C26—H26	119.4
C12—C11—C16	103.2 (2)	C25—C26—H26	119.4
C10—C11—H11	111.2	C26—C27—C28	117.9 (3)
C12—C11—H11	111.2	C26—C27—H27	121.1
C16—C11—H11	111.2	C28—C27—H27	121.1
C17—C12—C13	101.3 (3)	C27—C28—C23	121.9 (2)
C17—C12—C11	101.3 (2)	C27—C28—S1	129.4 (2)
C13—C12—C11	108.0 (3)	C23—C28—S1	108.56 (18)
C17—C12—H12	114.9		
			
C28—S1—N4—C22	1.39 (19)	C17—C15—C16—C9	−77.6 (3)
C7—C1—C2—C8	−63.5 (3)	C14—C15—C16—C9	175.6 (3)
C6—C1—C2—C8	173.9 (2)	C17—C15—C16—C11	34.9 (3)
C7—C1—C2—C3	173.3 (2)	C14—C15—C16—C11	−71.8 (3)
C6—C1—C2—C3	50.7 (3)	C10—C11—C16—C9	−2.1 (3)
C1—C2—C3—C4	−55.1 (3)	C12—C11—C16—C9	118.5 (2)
C8—C2—C3—C4	179.9 (3)	C10—C11—C16—C15	−120.4 (2)
C2—C3—C4—C5	58.4 (4)	C12—C11—C16—C15	0.2 (3)
C3—C4—C5—C6	−57.7 (4)	C14—C15—C17—C12	55.5 (3)
C4—C5—C6—C1	56.5 (4)	C16—C15—C17—C12	−55.8 (3)
C2—C1—C6—C5	−52.7 (3)	C13—C12—C17—C15	−55.7 (3)
C7—C1—C6—C5	−175.3 (2)	C11—C12—C17—C15	55.5 (3)
C9—N1—C7—C1	94.0 (3)	C8—N2—C18—C19	−177.22 (19)
C10—N1—C7—C1	−90.2 (3)	C21—N2—C18—C19	−52.0 (2)
C2—C1—C7—N1	171.9 (2)	C22—N3—C19—C18	154.8 (2)
C6—C1—C7—N1	−65.3 (3)	C20—N3—C19—C18	−63.5 (3)
C18—N2—C8—C2	−75.0 (2)	N2—C18—C19—N3	57.8 (2)
C21—N2—C8—C2	159.46 (19)	C22—N3—C20—C21	−155.6 (2)
C1—C2—C8—N2	136.8 (2)	C19—N3—C20—C21	62.0 (2)
C3—C2—C8—N2	−98.9 (3)	C18—N2—C21—C20	51.2 (2)
C10—N1—C9—O1	−179.0 (3)	C8—N2—C21—C20	177.88 (18)
C7—N1—C9—O1	−2.8 (4)	N3—C20—C21—N2	−55.6 (2)
C10—N1—C9—C16	2.6 (3)	S1—N4—C22—N3	172.05 (17)
C7—N1—C9—C16	178.8 (2)	S1—N4—C22—C23	−1.0 (3)
C9—N1—C10—O2	176.6 (3)	C20—N3—C22—N4	−12.3 (3)
C7—N1—C10—O2	0.4 (4)	C19—N3—C22—N4	126.7 (2)
C9—N1—C10—C11	−4.1 (3)	C20—N3—C22—C23	160.2 (2)
C7—N1—C10—C11	179.8 (2)	C19—N3—C22—C23	−60.7 (3)
O2—C10—C11—C12	70.7 (4)	N4—C22—C23—C28	0.0 (3)
N1—C10—C11—C12	−108.6 (3)	N3—C22—C23—C28	−172.9 (2)
O2—C10—C11—C16	−177.0 (3)	N4—C22—C23—C24	174.9 (2)
N1—C10—C11—C16	3.7 (3)	N3—C22—C23—C24	2.1 (4)
C10—C11—C12—C17	78.0 (3)	C28—C23—C24—C25	−2.4 (4)
C16—C11—C12—C17	−35.0 (3)	C22—C23—C24—C25	−177.0 (2)
C10—C11—C12—C13	−176.0 (2)	C23—C24—C25—C26	0.8 (4)
C16—C11—C12—C13	71.0 (3)	C24—C25—C26—C27	1.3 (4)
C17—C12—C13—C14	35.3 (4)	C25—C26—C27—C28	−1.8 (4)
C11—C12—C13—C14	−70.7 (3)	C26—C27—C28—C23	0.1 (4)
C12—C13—C14—C15	−0.6 (4)	C26—C27—C28—S1	175.9 (2)
C13—C14—C15—C17	−34.5 (3)	C24—C23—C28—C27	1.9 (4)
C13—C14—C15—C16	72.4 (3)	C22—C23—C28—C27	177.6 (2)
O1—C9—C16—C15	−67.3 (4)	C24—C23—C28—S1	−174.64 (18)
N1—C9—C16—C15	111.0 (3)	C22—C23—C28—S1	1.0 (2)
O1—C9—C16—C11	−178.5 (3)	N4—S1—C28—C27	−177.6 (2)
N1—C9—C16—C11	−0.1 (3)	N4—S1—C28—C23	−1.39 (19)

### Hydrogen-bond geometry (Å, º)

Cg is the centroid of the S1/ N4/C22/C23/C28 ring.

**Table:**

*D*—H···*A*	*D*—H	H···*A*	*D*···*A*	*D*—H···*A*
N2—H2*A*···Cl1	0.80 (2)	2.15 (2)	2.9426 (19)	168 (2)
C21—H21*A*···O1^i^	0.97	2.38	3.289 (3)	156
C5—H5*A*···*Cg*^ii^	0.97	2.89	3.802 (4)	157

Symmetry codes: (i) *x*+1/2, −*y*+3/2, −*z*+1; (ii) −*x*+1/2, −*y*+1, *z*−1/2.
